# 2456 Cutaneous lupus erythematosus patients have increased circulating myeloid-derived suppressor cells with immunosuppressive properties

**DOI:** 10.1017/cts.2018.57

**Published:** 2018-11-21

**Authors:** Stephanie Florez-Pollack, Lin-chiang Tseng, Masato Kobayashi, Benjamin F. Chong, Kiyoshi Ariizumi

**Affiliations:** Department of Dermatology, University of Texas Southwestern Medical Center, Dallas, TX, USA

## Abstract

OBJECTIVES/SPECIFIC AIMS: MDSCs are potent suppressors of T cell function, and have been recently found to be implicated in skin diseases driven by T cell dysregulation. However, the function of MDSCs in CLE is poorly understood. We sought to characterize the MDSC population in the peripheral blood of DLE patients and evaluate their ability to suppress autologous T cells. METHODS/STUDY POPULATION: All patients were recruited through the UT Southwestern Cutaneous Lupus Registry. PBMCs from 32 CLE patients and 16 age-matched and gender-matched controls were analyzed using flow cytometry. Monocytic MDSCs were identified by the phenotype of CD14+ HLA-DR neg/low. Furthermore, autologous MDSCs and T cells were purified from CLE PBMCs (n=4) and co-cultured at different ratios of these cells. T cell function was measured by secretion of IFN-γ by ELISA. RESULTS/ANTICIPATED RESULTS: Monocytic MDSCs in CLE PBMCs (median: 2.04%, IQR: 0.67%–5.07%) were significantly higher compared with healthy control PBMCs (median: 0.5%, IQR: 0.1%–1.07%, *p*=0.002). Although not significant on subset analysis, patients with CLE limited to the head and neck had the highest levels of MDSCs. CLE MDSCs (n=4) were found to suppress autologous activated T-cells in a dose-dependent manner. DISCUSSION/SIGNIFICANCE OF IMPACT: In this cross-sectional study of patients of the UT Southwestern Cutaneous Lupus Registry, we observed differences in the levels of MDSCs among PBMCs of CLE patients Versus healthy controls. CLE patients had significantly higher levels of MDSCs, which could be explained by the presence of an inflammatory state in this group. Furthermore, CLE MDSCs were able to suppress autologous T cells, showing that these cells are functionally patent in CLE blood. Their up-regulation in CLE blood may represent the body’s response to limiting disease severity, since most patients had mild disease activity.

